# Fabrication of hydroxyapatite coatings on AZ31 Mg alloy by micro-arc oxidation coupled with sol–gel treatment

**DOI:** 10.1039/c7ra10951b

**Published:** 2018-04-03

**Authors:** Hui Tang, Wei Tao, Chao Wang, Huilong Yu

**Affiliations:** School of Materials and Energy, University of Electronic Science and Technology of China Chengdu 611731 China tanghui@uestc.edu.cn; Shanxi Key Laboratory of Advanced Magnesium-Based Materials, Taiyuan University of Technology Taiyuan 030024 P. R. China; Department of Electrical and Computer Engineering, University of Houston Houston Texas 77204 USA; Institute of Machinery Manufacturing Technology, China Academy of Engineering Physics Mianyang 621900 Sichuan China; School of Chemical Engineering and Technology, Harbin Institute of Technology Harbin 150001 China; Institute of Materials, China Academy of Engineering Physics P. O. Box No. 9-12, Huafengxincun Jiangyou City Sichuan Province 621908 P. R. China yuhuilong2002@126.com

## Abstract

Magnesium (Mg) alloys, can potentially be used as biodegradable orthopedic implants because of their biodegradability and good mechanical properties. However, a quick degradation rate and low bioactivity have prevented their clinical application. In order to enhance the corrosion resistance and the *in vitro* bioactivity of Mg alloys, protective composite coatings were prepared on AZ31 magnesium alloy followed by sol–gel sealing treatment under low-pressure conditions. The morphologies, crystalline structure and the composition of the samples were characterized by SEM, XRD, and XPS. Electrochemical corrosion test and the *in vitro* bioactivity were also studied. The results indicated that the composite coatings not only improved the corrosion resistance, but also enhanced the *in vitro* bioactivity of AZ31 Mg alloy. Therefore, Mg alloy treated with micro-arc oxidation and sol–gel offers a promising approach for biodegradable bone implants.

## Abstract 2

Magnesium (Mg) alloys, can potentially be used as biodegradable orthopedic implants because of their biodegradability and good mechanical properties.

## Graphical abstract

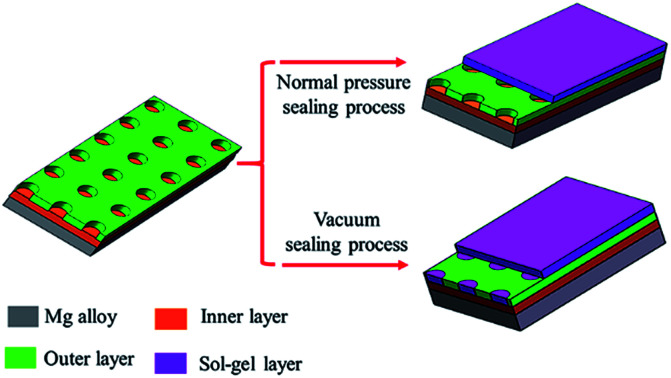

## 1. Introduction

In recent years, utilization of magnesium and its alloys as potential candidates for bone implants has drawn increasing attention, owing to their mechanical match with living bone and gradual degradation in the human physiological environment. However, the rapid corrosion rate in the physiological environment has become the main drawback, which is hindering their applications.^1–4^ The rapid corrosion of magnesium alloys results in the loss of their mechanical integrity before sufficient bone tissues are recovered. Moreover, rapid corrosion of Mg results in many other adverse effects, including the formation of hydrogen gas cavities and increase of local pH, which is harmful to tissue healing.^5–7^ Therefore, in order to obtain wide clinical practice, it is necessary to improve the corrosion resistance of magnesium alloys. Surface modification is considered as an effective way to improve the corrosion resistance and the biocompatibility of magnesium alloys. Alkaline heat treatment,^8^ electrodeposition,^9^ conversion treatment,^10^ and micro-arc oxidation,^11^ have been used to improve the corrosion resistance of Mg alloys in recent years.

In all kinds of magnesium alloy surface modification methods, micro-arc oxidation (MAO) is recognized as a promising and effective technique. This is due to the fact that it can introduce firmly adherent ceramic coatings onto the surface of magnesium alloys, which could significantly improve the hardness, wear resistance and corrosion resistance of Mg alloys.^12,13^ Unfortunately, large number of micro-pores and micro-cracks is present on the MAO coating, which permits corrosive intermedium to be absorbed into the coating and decrease the corrosion resistance of MAO coating on magnesium.^14^ To further improve the protective properties of MAO coating on Mg alloys, remediation of the surface defects of MAO coating was considered as a significant approach.^15,16^ Moreover, as the main component of the MAO coating, magnesium oxide (MgO) is lack of biological activity.^17^ Therefore, researchers are making efforts to fabricate biological layers improve the corrosion resistance and the biocompatibility of the MAO coating.^18,19^ Hydroxyapatite (HA) is the main inorganic component of the bone tissue and has similar chemical and biological characteristics to human bone tissue as well as its ability to directly bond to the surrounding tissues.^20^ A number of techniques, such as electrophoretic deposition,^21^ chemical deposition,^22^ and hydrothermal treatment^23^ have been developed to form an HA layer on the surface of the MAO coating. However, most of these techniques employed only formed an additional barrier layer on the surface of the MAO coating, failing to fill the micro-pores and micro-cracks in MAO coating. Sol–gel process has attracted much attention to seal the pores of MAO coating because it is environmentally friendly and is capable of reaching homogeneous composition.^24^ But under the atmospheric pressure, the sol is difficult to fill the micro-pores and micro-cracks, because of the tendency to hold micro air bubbles in these defects.

In this study, the sol–gel technique was utilized to seal the micro-pores and micro-cracks present in the MAO coating. Low-pressure were employed to remove micro air bubbles, allowing sol enter the micro-pores and micro-cracks. The effects of sintering temperature on the surface morphology, composition, biological activity and corrosion resistance of the composite coatings were investigated systematically.

## 2. Experiment procedure

### 2.1 Preparation of MAO coating

A commercial AZ31 magnesium alloy (3.0–3.4 wt% Al, 0.8 wt% Zn, 0.4 wt% Mn, balance Mg) was machined to obtain the substrates of the size 10 × 10 × 1 mm. The surface of the substrates was grinded with SiC abrasive papers of up to grit 600, followed by ultrasonic cleaning in acetone for 20 min.

The MAO treatment was carried out using a HIT-4C power supply. The AZ31 sample and a stainless steel plate were used as the anode and cathode, respectively. The electrolyte for the MAO process was composed of 4 g L^−1^ sodium hydroxide and 6.3 g L^−1^ calcium glycerophosphate (CaGP). The MAO process was carried out at a constant voltage of 400 V and frequency of 1000 Hz for 40 min, and the temperature of the electrolyte was kept below 30 °C with a cooling system.

### 2.2 Preparation of sol–gel after the MAO process

Preparation of the HA sol was performed using the following method. Triethylphosphite ((C_2_H_5_O)_3_P) and calcium nitrate (Ca(NO_3_)_2_) were selected as the source for P and Ca, respectively. Triethylphosphite diluted with anhydrous ethanol was firstly hydrolyzed for 12 h with distilled water. A stoichiometric amount of 2 M calcium nitrate dissolved in anhydrous ethanol was then added to the hydrolyzed phosphite sol which had previously been aged for 24 h (molar ratio Ca : P = 1.67). The mixed solution was stirred for 4 h and aged for 3 days at room temperature to obtain the fully aged HA sol.

The sealing treatment was carried out under vacuum condition. The sample with MAO coating was placed in a filter flask. A separatory funnel filled with HA sol was mounted on the cap of the filter flask. Vacuum was applied by the pump to the filter flask, thus removing the air pockets from the MAO pores. HA sol was then introduced into the filter flask. The composite samples were firstly dried at 60 °C, and then heat treated at a temperature of 100 °C, 200 °C or 400 °C. In order to seal most of the pores, this process was repeated twice. The MAO and the composite-coated samples are noted as MAO, MAO-H1, MAO-H2, and MAO-H4, based on the heat treatment temperature.

### 2.3 Characterization of MAO coatings

The surface morphology, cross-section and elemental distribution of the coatings were examined by scanning electron microscopy (SEM, Hitachi High-Technologies Corp., S-4800) and energy dispersive X-ray spectrometry (EDS, Oxford instruments X-Max, England). The phases of the samples were characterized by X-ray diffraction (XRD, Rigaku Dymax, Japan) using Cu Kα radiation and a monochromator at 40 kV, 200 mA with a scanning rate and a step of 4° min^−1^. Surface chemistry of the samples was analyzed by X-ray photoelectron spectroscopy (XPS) with a monochromatic Al Kα source. The electron take-off angle was fixed at 45° and the vacuum pressure was less than 10^−7^ torr during data acquisition.

### 2.4 Bonding strength tests

The tensile adhesion strength between the coating and the substrate was measured using a universal testing machine, according to the modified ASTMC-633.^25^ One side of the coating was grinded using sandpaper. Parallel aligned cylinders were glued to both the coated and the back sides of the specimens with epoxy resin. And five adhesion strength tests were performed for each sample.

### 2.5 Electrochemical tests

The coated specimens were molded into epoxy resin with only one side of 1 cm^2^ exposed for the electrochemical test. The electrochemical tests were carried out at 37 ± 1 °C of Hank's solution using a PARSTAT 2273 automatic laboratory corrosion measurement system. Hank's solution was prepared by dissolving analytical-grade chemicals of 8 g L^−1^ NaCl, 0.4 g L^−1^ KCl, 0.35 g L^−1^ NaHCO_3_, 0.25 g L^−1^ NaH_2_PO_4_·H_2_O, 0.06 g L^−1^ Na_2_HPO_4_·2H_2_O, 0.19 g L^−1^ CaCl_2_·2H_2_O, 0.19 g L^−1^ MgCl_2_, 0.06 g L^−1^ MgSO_4_·7H_2_O and1 g L^−1^ glucose. The process was conducted using a conventional three-electrode electrochemical cell with a saturated calomel electrode (SCE) as the reference electrode, a platinum plate as the counter electrode and the sample as the working electrode. Prior to testing, each sample was kept in Hank's solution for 30 min to reach a steady open circuit potential value. The potentiodynamic polarization curves were measured from −0.25 to 0.25 V with respect to the open circuit potential at a scan rate of 1 mV s^−1^. The electrochemical impedance spectroscopy (EIS) test was gauged from 100 kHz to 0.01 Hz with 10 points per decade. The amplitude of the sinusoidal potential was 10 mV with respect to the OCP. All EIS data were analyzed using the software Zview.

### 2.6 *In vitro* bioactivity test

The *in vitro* reactivity study was carried out by soaking the samples into the SBF solution at 37 °C at with the variation of immersion time. The SBF was prepared by dissolving reagent grade chemicals of NaCl, NaHCO_3_, KCl, K_2_HPO_4_·3H_2_O, MgCl_2_·6H_2_O, CaCl_2_ and Na_2_SO_4_ sequentially into distilled water and buffering at pH 7.40 with tris(hydroxymethyl)aminomethane and dilute HCl at 37 °C. The ionic concentrations of Na^+^, K^+^, Mg^2+^, Ca^2+^, Cl^−^, HCO_3_^2−^, HPO_4_^2−^ and SO_4_^2−^ of SBF were 142.0, 5.0, 1.5, 2.5, 147.8, 4.2, 1.0 and 0.5 mmol L^−1^, respectively. The solutions were renewed every other day. After soaking for the pre-determined time period, the samples were removed from the solution, gently rinsed with distilled water and dried at room temperature. The morphological and elemental analyses of the coatings were performed by SEM/EDX. The phases present in the coating were determined by Shimadzu 6000 Lab XRD system.

## 3. Results and discussion

**Fig. 1:**
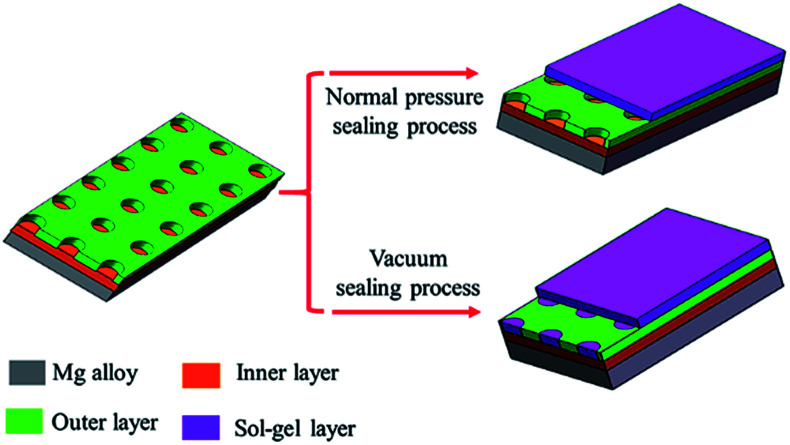
Scheme of sealing process under normal pressure and vacuum.

Sol–gel layer sealing the pores of MAO coating opens a new approach to improve the corrosion resistance of MAO coated Mg alloys.^26,27^ Sol–gel layer dispersed on the surface of MAO coating is expected to enter into the pores, and thus constrain the pass-through of the corrosive ions. However, the sealing process by sol under normal pressure lead to poor sealing effect mainly because the sol hard to enter into the pores due to the blocking effects of the air in the pores. Herein, we report a vacuum method to seal the pores for improving the corrosion behavior of the MAO coatings. Sealing under normal pressure, the sol is hard to enter into the pores and only form a gel layer on the surface of the MAO coating. Sealing under vacuum condition, the sol can enter the pores and deposit onto the pores due to the lower pressure in the pores.^28^ So the gel fills the pores and a gel layer forms on the surface of the MAO coating. The scheme of these two approaches is showing in Fig. 1.

**Fig. 2:**
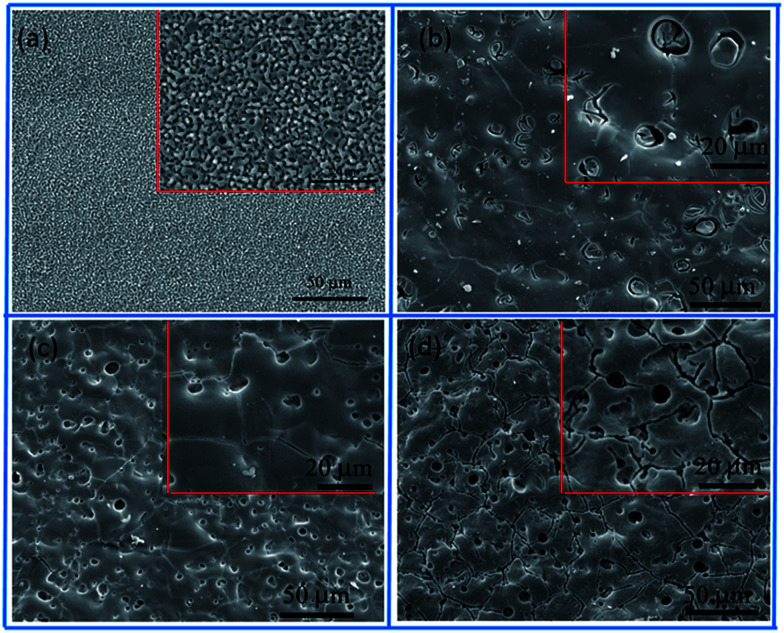
SEM micrographs of (a) MAO, (b) MAO-H1, (c) MAO-H2 and (d) MAO-H4.

The surface morphologies of the MAO coating and HA sol–gel sealed composite coatings are shown in Fig. 2. The typical porous microstructure can be observed on the MAO coated Mg alloy, which is consistent with other works.^29^ The average diameter of the pores was estimated to be between 1 to 3 μm. It was reported that the pores were formed by the molten oxide and gas bubbles thrown when exiting from the micro-arc discharge channels.^30^ The pores permit the penetration of the corrosive medium to the Mg alloy substrate and allow corrosion to proceed. To reduce the corrosion rate, it is necessary to seal these pores. Fig. 2(b)–(d) reveals the surface morphologies of the MAO samples after sol–gel treatment. Fig. 2(b) shows the surface of the sol–gel layer on MAO coating which was heat treated at 100 °C. The sol–gel layer uniformly covered on the surface of the MAO coating, and sealed most of its pores. Some gaps can be observed in pores, which could be attributed to the shrinkage of sol–gel during heat treatment. SEM observations indicate that the surface of the layer was homogeneous and dense in appearance and that no cracks could be found in the layer from heat treatment at 200 °C, as shown in Fig. 2(c). Fig. 2(d) reveals the surface of sol–gel layer treated at 400 °C. Some tiny cracks and pores were visible on the surface of the sol–gel layer. Pores and cracks were considered common in the sol–gel layer, reported by previous researchers.^31^ As the composite coating on AZ31 Mg alloy is dried at 100 °C, and a dried gel layer is formed. When the dried gel layer is heated, residual organics decompose and the layer densities.^32,33^ It is supposed that these cracks and pores are generated during the heat-treatment process.^34^ In the case of HA layer fabricated on the surface of the MAO coating at low temperature, the HA sol goes into the pores and forms a layer on the surface. During the heat-treatment process, the fine pores and cracks are produced ascribed to the decomposing of the organic and the stress produced at an elevated temperature resulting from the discrepancy between the coating and the substrate in terms of their thermal expansion coefficient.^35^ The amount and the size of cracks in the sol–gel layer depends on many factors such as the heating temperature, the thickness, and compactness of the layer. However, these tiny pores and cracks compared the pores on the surface of the MAO coating are insignificant.

**Fig. 3:**
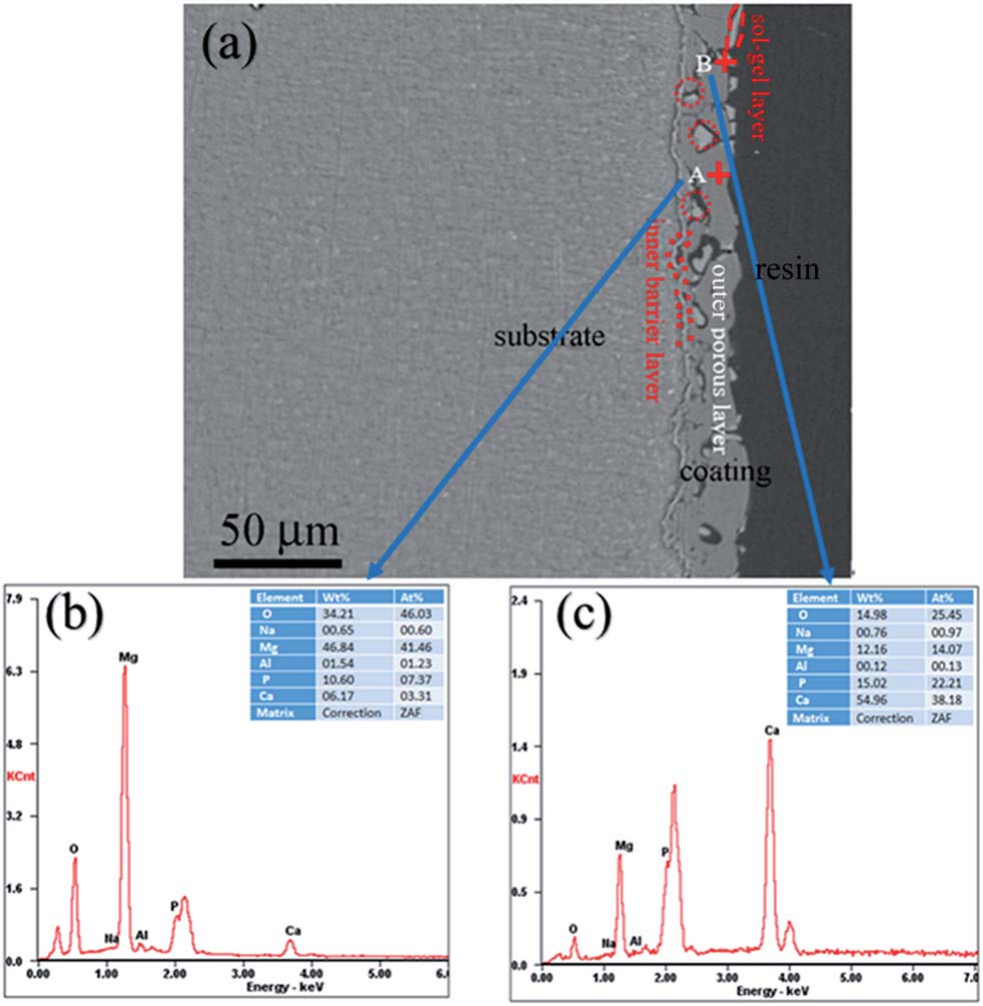
(a) Cross-section morphology of the MAO-H2, (b) analysis on point A, and (c) EDS analysis on point B.

Fig. 3 shows the cross-sectional morphology of the composite coating treated at 200 °C and its EDS analyses. As shown in Fig. 3(a), the composite coating is composed of sealing layer, the outer porous layer, and the inner barrier layer. The sealing layer of less than 1 μm is connected to the MAO coating by physical interlocking. Some part of the sealing layer was exfoliated, which may result from the shrinkage stress of the curing resin during the cross-sample preparation. It can be clearly observed that sealing agent can penetrate into and locked into most pores (the red circle in Fig. 3(a)). Composition analysis was performed using EDS. The EDS spectra of points A and B are shown in Fig. 3(b) and (c). Point A is part of the MAO coating. While point B is the sealing agent, which was located in the pores of the MAO coating. EDS analysis of point A exhibits the presence of Mg, O, Al, Na, Ca and P. The presence of Mg and Al elements imply that the substrate elements entered into the coating during the MAO process. Elements of Ca and P appearance at point A may come from the electrolyte during the MAO process. EDS analysis performed on point B also reveals the presence of Mg, O, Al, Na, Ca and P. However, it shows the lower concentration of Mg and higher concentrations of Ca and P when compared with that of point A. The atomic ratio of Ca/P is about 1.72, which is much higher than that of point A, and is similar to the ratio of Ca/P in the sol–gel. As a result, it can be concluded that the sealing agent can penetrate into the pores, and the pores in the MAO coating provided much larger contact areas between MAO coating and sol–gel layer, which resulted in a much enhanced bonding strength.

**Fig. 4:**
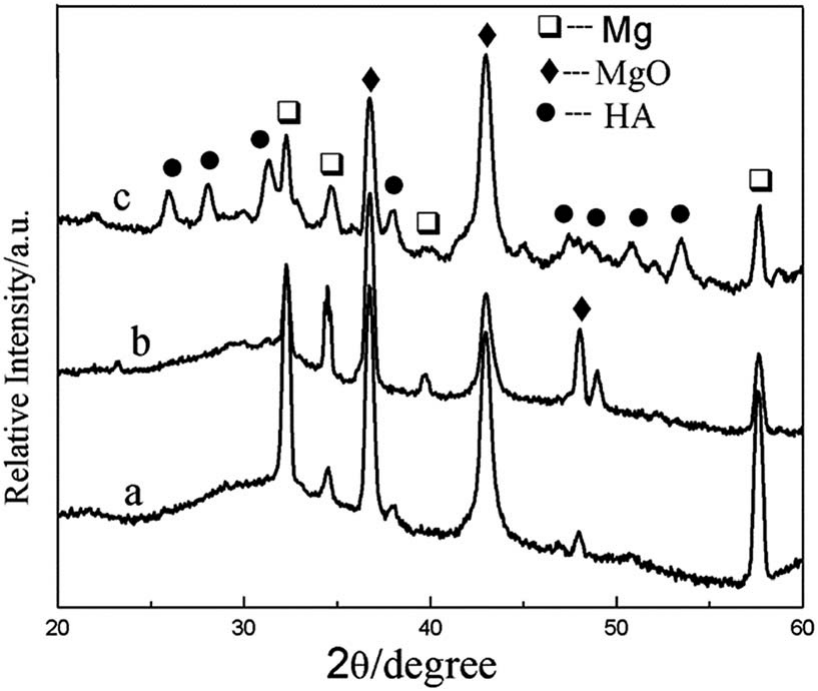
XRD patterns of the composite coatings of (a) MAO-H1, (b) MAO-H2, and (c) MAO-H4.

XRD patterns of the composite coatings produced at different heat treatment temperatures are given in Fig. 4. When the heat treatment temperature is 100 or 200 °C, XRD analysis shows that the phase of the coating is mainly MgO. After heat treated at 400 °C, the HA diffraction peaks appeared, which indicates that Ca and P in the composite has transformed into HA. It showed be noted that the transformation temperature is much lower than previously reported.^36^ The crystallization temperature of HA is higher than 800 °C, when HA was synthesized by the sol–gel. In this study, it appears that the crystallization temperature of HA is lower than 400 °C, which may be attributed to the crystal transformation of Ca and P in the MAO coating.

**Fig. 5:**
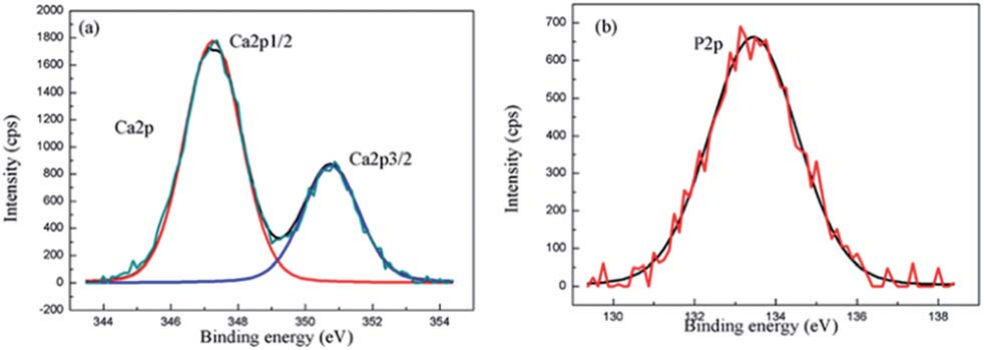
XPS spectra of the MAO-H4 (a) Ca2p, and (b) P2p.

In order to identify the chemical state of Ca and P in the composite coating, the XPS analysis of the composite coating for Ca2p and P2p was performed. The Ca2p spectrum consists of two peaks at the binding energies of 347.8 eV for Ca2p3/2 and 351.4 eV for Ca2p1/2 (Fig. 5(a)). The Ca2p peaks in the XPS spectrum are in accordance with the binding energies of calcium in the HA structure.^37^ The P2p level at the binding energy of 133 eV as shown in Fig. 5(b), asserts the existence of PO_4_^3−^ in the coating.^38^ The XPS results confirm that the surface layer contains HA.

**Fig. 6:**
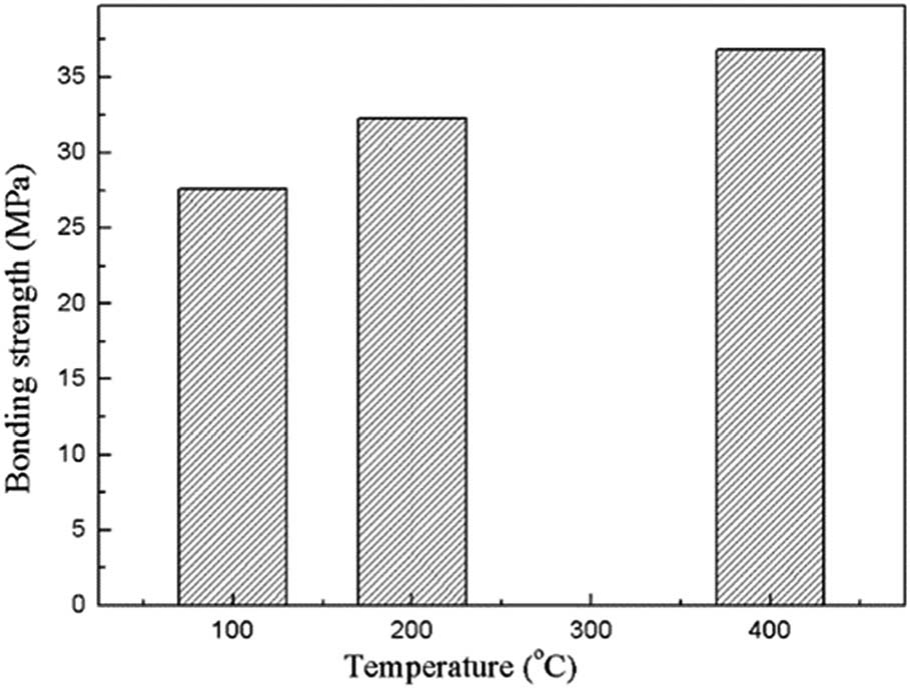
Bonding strengths of the composite coatings prepared under different heat treatment temperatures.

Adhesion of the composite coating with the magnesium substrate is one of the most important characteristics for the *in vivo* implantation. The bonding strength of the composite coatings prepared under various heat treatment temperatures is showing in Fig. 6. It indicates that the bonding strengths of the composite coatings are higher than 25 MPa, and shows a significant increase in the increasing heat treatment temperature. The high bonding strength is achieved due to the insertion of the MAO coating. The porous outer layer of the MAO coating locked with the sol–gel layer due to the much enhanced surface contact area. They bonded together in a much stronger manner owing to the heat treatment. As a matter of fact, bonding the sol–gel coating directly on bare Mg alloy was also attempted in the present study, but the coating was easily detached from the substrate. As a result, the value of bonding strength between the sol–gel layer and bare Mg alloys was difficult to be tested out.

**Fig. 7:**
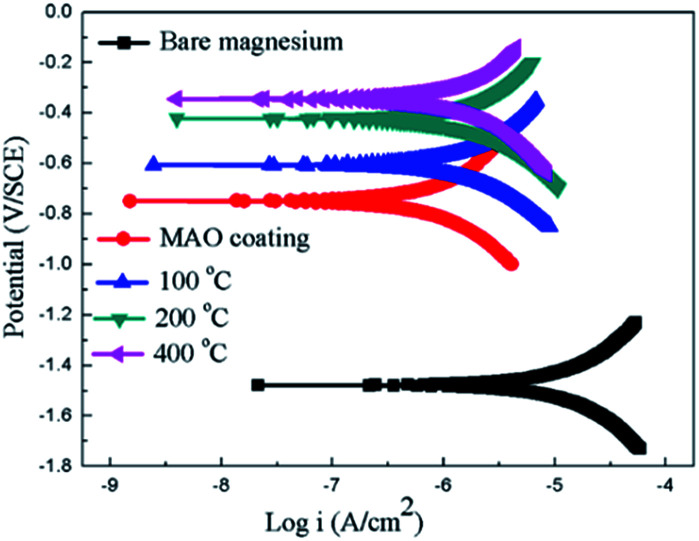
Potentiodynamic polarization curves for the coated and uncoated samples.

The potentiodynamic polarization curves of the bare Mg alloy, MAO and the composite coating samples tested in Hank's solution are shown in Fig. 7. The corrosion potential (*E*_corr_) and corrosion current density (*I*_corr_) evaluated according to Tafel extrapolation and were listed in Table 1. Overall, the MAO and the composite coated samples show higher corrosion potential and lower corrosion current density than bare Mg alloy. The corrosion potential of bare Mg alloy was as low as −1.45 V, while the MAO coated dramatically increased its corrosion potential up to −0.75 V. The corrosion current decreased from 1.05 × 10^−5^ A cm^−2^ to 5.21 × 10^−6^ A cm^−2^, which illustrates that the MAO coating can enhance the corrosion resistance of the Mg alloy. After sol–gel treatment, the composite coated samples showed further improvement in terms of the corrosion resistance of bare Mg alloy. The corrosion potentials of the composite coated samples were much higher than that of the MAO coated sample, and the composite samples showed even lower corrosion current density than that of the MAO coated sample. This can be attributed to the microstructural difference between the MAO coating and the composite coatings. A large number of micro-pores existed in the MAO coating, which allowed the passage of the corrosive ions, resulting in decreased corrosion resistance. As to the sample with composite coatings, the micro-pores filled with the sol–gel, which effectively inhibited the penetration of the corrosive ions into the composite coating. The corrosion potential of the composite samples shifted more positively with the increase of the heat treatment temperature. And the corrosion current density shifted to lower values as heat treatment temperature increased. Among the composite coated samples, the MAO-H4 sample showed the lowest corrosion current and most positive corrosion potential.

**Table 1:** Summary of the corrosion potential voltage and the current density of the uncoated and coated samples

Samples	*E*_corr_ (V)	*I*_corr_ (A cm^−2^)
Sub	−1.45	1.05 × 10^−5^
MAO coating	−0.79	5.21 × 10^−6^
MAO-H1	−0.66	7.13 × 10^−7^
MAO-H2	−0.43	6.27 × 10^−7^
MAO-H4	−0.37	4.52 × 10^−7^

**Fig. 8:**
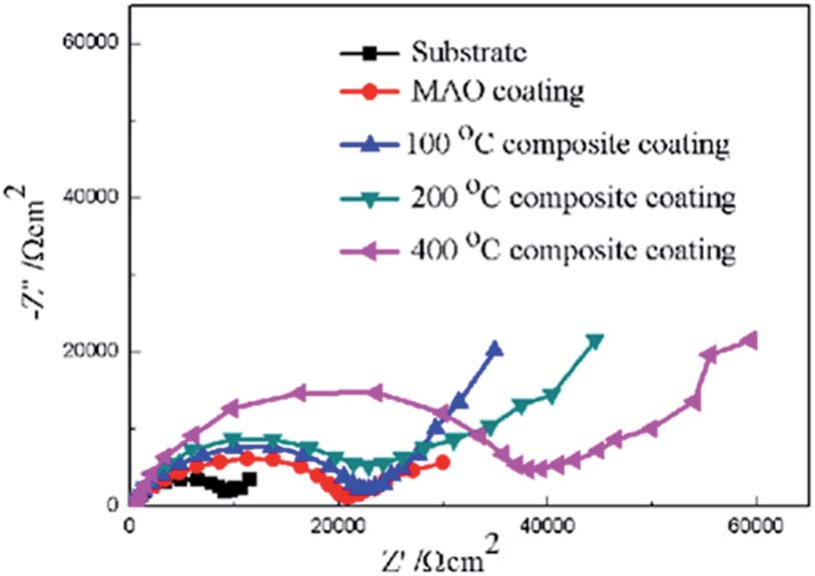
Nyquist plots of for the coated and uncoated samples.

**Fig. 9:**
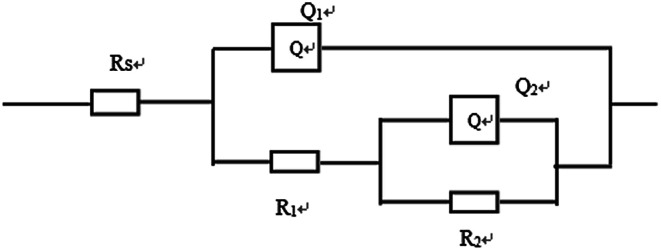
Electrical equivalent circuit used for fitting the experimental EIS spectra.

Furthermore, electrochemical impedance spectroscopy (EIS) was employed to evaluate the corrosion behaviors of the bare Mg alloy, MAO and composite coated samples in Hank's solution. Nyquist plots of these samples are shown in Fig. 8. In the Nyquist plot, the coated samples exhibit two capacitive loops, which correspond to the two-layer structure of the MAO coating and the composite coatings, *i.e.*, the outer porous layer and the inner compact layers. The time constant appearing in the high-frequency range reflects the properties of the outer porous layer, while the constant appearing in the low-frequency range characterizes the properties of the inner compact layer. As the structure shown in Fig. 2(a), the porous layer of the MAO coating provides a good surface for the deposition of the sol–gel layer, with the sol–gel being locked into the pores. So the sol–gel layer and the outer porous layer should be regarded as a whole layer against the corrosion attack. According to the curves shown in Fig. 8 and the special structure of the coatings, the equivalent circuit is proposed as shown in Fig. 9. In the equivalent circuit, the *R*_s_ is the solution resistance, *R*_p_ and *Q*_p_ are suggested to represent the resistance and constant phase element of the porous layer. *R*_b_ is the barrier layer resistance of the treated coating in parallel with *Q*_b_. It was reported that a corrosion product layer was formed when Mg substrate is immersed in the corrosive solution.^39^ The equivalent circuit is shown in Fig. 9, can also be used to fit the corrosion process of the Mg alloy. The *R*_p_ is suggested to represent the resistance of the coating in the form of corrosion product, and *R*_b_ associated with the charge transfer process at the metal/electrolyte interface. The corresponding EIS data are presented in Table 2. It can be observed that the *R*_s_ value is very low for the tests because Hank's solution is very conductive. The EIS results indicate that the impedance of bare the Mg sample is very low. The MAO coated sample has much higher resistance than bare Mg, which suggests that the MAO coating protected the bare Mg. *R*_b_ of the MAO coating is much higher than *R*_p_, which is because the outer layer of the MAO coating had many pores and permitted the penetration of the corrosive ions. For the composite coatings, due to the covering and sealing of the sol–gel layer, the transfers of corrosive ions were significantly inhibited, resulting in much higher *R*_p_ values when compared with that of the MAO coating. This is accordance with the results from the examination of the surface and cross-section morphologies as shown in Fig. 2 and 3, which clearly illustrated the reduction of the number of the pores after the sol–gel treatment. As seen from Table 2, the *R*_b_ value of the composite coatings is higher than that of the MAO coatings, indicating that the performance of the inner layer of the MAO coating was improved by the sol–gel treatment to a certain extent. This means that the sol–gel treatment provided better protection for the Mg alloy against corrosion. It can also be seen that the values of *R*_p_ and *R*_b_ increase with the increasing heat treatment temperature, which is attributed to the chemical composition and structural difference. The results of the potentiodynamic polarization and electrochemical impedance spectroscopy tests imply that the sol–gel deposited into the pores and formed a sol–gel layer on the surface of MAO coating, which enhanced the corrosion resistance of the Mg alloys.

**Table 2:** Results of the EIS data fitting by the electrical equivalent circuit

Samples	*R*_s_ (Ω cm^2^)	*Q*_1−*Y*_0__ (Ω^−1^ cm^−2^ s^−*n*^)	*Q*_1−*n*_	*R*_p_ (Ω cm^2^)	*Q*_2−*Y*_0__ (Ω^−1^ cm^−2^ s^−*n*^)	*Q*_2−*n*_	*R*_b_ (Ω cm^2^)
Sub	4.7 × 10^−2^	5.21 × 10^−8^	0.9	672	4.31 × 10^−8^	0.9	1566
MAO	1.3 × 10^−3^	8.62 × 10^−7^	0.8	3655	7.12 × 10^−7^	0.7	9271
MAO-H1	2.9 × 10^−3^	2.56 × 10^−7^	0.7	1.03 × 10^4^	1.03 × 10^−7^	0.8	1.16 × 10^4^
MAO-H2	3.8 × 10^−2^	6.73 × 10^−7^	0.8	1.55 × 10^4^	6.38 × 10^−7^	0.8	1.21 × 10^4^
MAO-H4	1.2 × 10^−3^	1.36 × 10^−7^	0.7	1.78 × 10^4^	8.12 × 10^−6^	0.7	1.16 × 10^4^

**Fig. 10:**
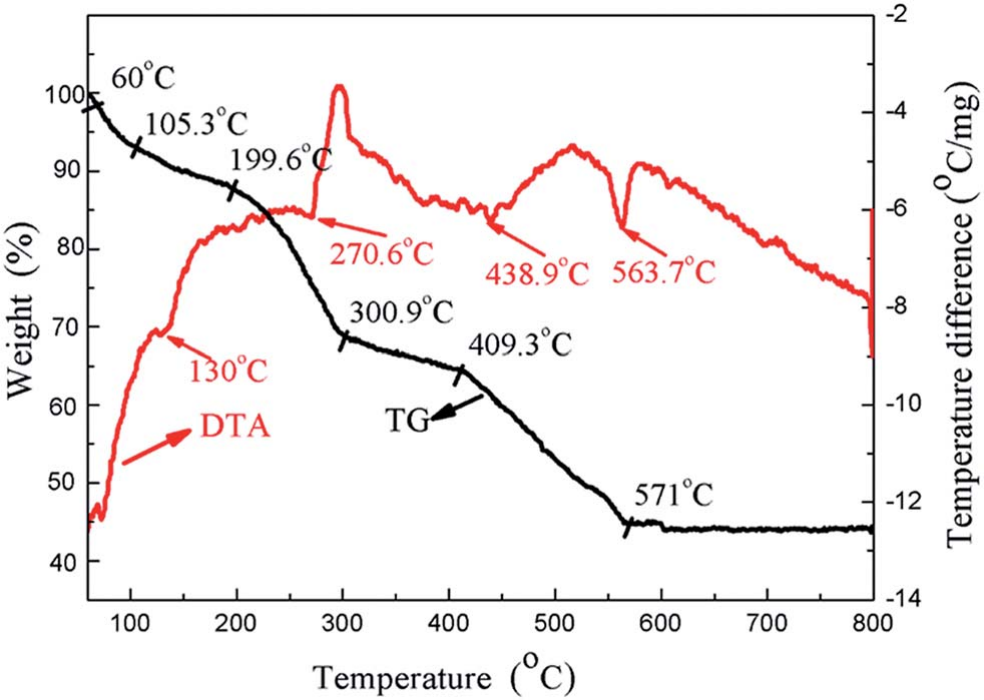
TG-DTA analysis of the HA gel from room temperature to 800 °C.

To further explore the effect of the heat treatment temperature, thermal gravimetric and differential thermal analyses (TG-DTA) were performed from room temperature up to 800 °C at a heating rate of 10 K min^−1^ for the HA gel (Fig. 10). It can be seen that four endothermal peaks appear in the whole temperature range. Three endothermic peaks appear at 130 °C, 270.6 °C, and 438.9 °C, which can be attributed to the decomposition of the nitrates and organic compounds.^40^ The endothermic peak at 563.7 °C is associated with the crystallization of HA.^41^ In the process of preparing the composite coating samples, the heat treatment temperatures were 100, 200 and 400 °C, which fall in the decomposition temperature ranges of the nitrates and organic compounds. Along with the decomposition of the nitrates and organic compounds, the compound of the gel becomes more stable, which is beneficial to the corrosion resistance. On the other hand, the bonding strength of the composite coating increases with the increasing heat treatment temperature, which also improves the corrosion resistance. As a result, the corrosion resistance of the composite coatings increases with the increasing heat treatment temperature.

**Fig. 11:**
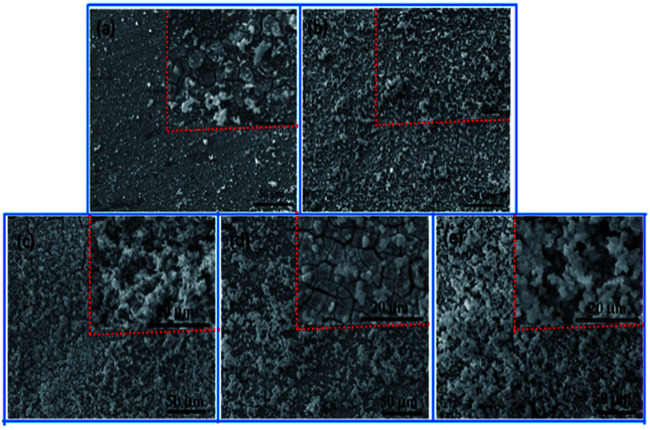
The SEM morphologies of AZ31, MAO, MAO-H1, MAO-H2 and MAO-H4 immersed in SBF for 72 h.

*In vivo* bioactivity of the coatings can be evaluated by soaking the sample in SBF and examining the formation of apatite. The microscopical surface morphologies of AZ31, MAO, MAO-H1, MAO-H2 and MAO-H4 samples after an immersion time of 72 h in the SBF are shown in Fig. 11. After 72 h immersion, many large and deep network-like cracks can be observed on the surface of the AZ31 sample due to the corrosion (Fig. 11(a)). A few white particles precipitated on the AZ31 surface. According to Fig. 11(b), the surface of the MAO sample remains porous after SBF immersion. Some apatite-like particles randomly scatter on the sample surface. For the MAO-H1 sample, the surface area was mostly covered by the spongy aggregation of spherical particles. The entire surface of MAO-H2 was covered by a layer of precipitates after immersion in SBF for 72 h. Micro-cracks spreads on this layer, which indicates the presence of a thicker apatite layer on this surface. For MAO-H4, the surface of the coating was covered by a newly formed layer consisting of small granular structures. Other authors also observed similar morphologies, the spherical particles and precipitate layer being reported as apatite.^42,43^ This suggests that AZ31, MAO, MAO-H1, MAO-H2 and MAO-H4 have the ability to induce the bone-like apatite nucleation and growth on their surfaces from SBF. It can also be concluded that the composite coating samples could introduce more precipitates onto the surface than bare AZ31 and MAO sample given the same immersion time.

The formation process of HA crystals was affected by two factors: nucleation of HA and diffusion of Ca and P from inner coating towards coating surface. HA nucleate on the surface of the samples, and they spontaneously grow and give rise to HA products according to the following reaction:^44^10Ca^2+^ + 6PO_4_^3−^ + 2OH^−^ ↔ Ca_10_(PO_4_)_6_(OH)_2_

It is reported that AZ31 Mg could induce the formation of HA in SBF solution because of the corrosion product of OH^−^. OH^−^ can absorb Ca^2+^ and PO_4_^3−^ ions into the surface. These induce over-saturation of OH^−^, Ca^2+^, and PO_4_^3−^ on the surface.^45^ Additionally, the MAO coating and the sol–gel layer can release calcium and phosphate ions on immersion in SBF to increase the degree of supersaturation. Once the apatite nuclei are formed, they can grow spontaneously by consuming the calcium and phosphate ions in the surrounding fluid. So apatite can be formed in a shorter time. That is to say, the film has bioactivity.

## 4. Conclusions

In this study, an effective method of sealing the pores of the MAO coating to improve the corrosion resistance and bioactivity of Mg alloy implants was reported. The composite coatings were obtained by sealing the pores under low-pressure conditions using the HA sol–gel process. The HA not only covered the surface of the MAO coating but also penetrated and locked into the pores. This resulted in the integration of the HA sol–gel layer with the MAO coating by physical interlocking. The HA sol–gel layer provided protection against corrosion by physically sealing the pores of the MAO coating and acting as a barrier. It significantly improved the corrosion resistance and bioactivity of the MAO coating. The corrosion resistance, along with the bonding strength between the composite coating and the Mg alloy increased with the increasing heat treatment temperature. This was attributed to the decomposition of the nitrates and organic compounds with increasing heat treatment temperature.

## Conflicts of interest

There are no conflicts to declare.

## Supplementary Material
